# Methylation of the *PTENP1* pseudogene as potential epigenetic marker of age-related changes in human endometrium

**DOI:** 10.1371/journal.pone.0243093

**Published:** 2021-01-22

**Authors:** Tatyana F. Kovalenko, Ksenia V. Morozova, Marat S. Pavlyukov, Ksenia S. Anufrieva, Mikhail Yu. Bobrov, Alina M. Gamisoniya, Lyudmila A. Ozolinya, Yulia E. Dobrokhotova, Mikhail I. Shakhparonov, Lev I. Patrushev

**Affiliations:** 1 Laboratory of membrane bioenergetics, Shemyakin–Ovchinnikov Institute of Bioorganic Chemistry Russian Academy of Sciences, Moscow, Russia; 2 Department of Obstetrics and Gynecology, Pirogov Russian National Research Medical University, Moscow, Russia; 3 Center for Precision Genome Editing and Genetic Technologies for Biomedicine, Federal Research and Clinical Center of Physical-Chemical Medicine of Federal Medical Biological Agency, Moscow, Russia; 4 Laboratory of Cell Biology, Federal Research and Clinical Center of Physical-Chemical Medicine of Federal Medical Biological Agency, Moscow, Russia; 5 Moscow Institute of Physics and Technology (State University), Moscow Region, Russia; 6 Laboratory of Molecular Pathophysiology, Kulakov Research Center of Obstetrics, Gynecology and Perinatology, Ministry of Healthcare of Russian Federation, Moscow, Russia; 7 Laboratory of oxylipins, Shemyakin–Ovchinnikov Institute of Bioorganic Chemistry Russian Academy of Sciences, Moscow, Russia; 8 Educational & scientific center, Shemyakin–Ovchinnikov Institute of Bioorganic Chemistry Russian Academy of Sciences, Moscow, Russia; Peking University Cancer Hospital and Institute, CHINA

## Abstract

The processed pseudogene *PTENP1* is involved in the regulation of the expression of the *PTEN* and acts as a tumor suppressor in many types of malignances. In our previous study we showed that *PTENP1* methylation is present not only in tumor, but also in normal endometrium tissues of women over 45 years old. Here we used methylation-specific PCR to analyze methylation status of CpG island located near promoter region of *PTENP1* in malignant and non-malignant endometrium tissues collected from 236 women of different age groups. To confirm our results, we also analyzed RNA sequencing and microarray data from 431 women with endometrial cancer from TCGA database. We demonstrated that methylation of *PTENP1* is significantly increased in older patients. We also found an age-dependent increase in the level of *PTENP1* expression in endometrial tissue. According to our data, *PTENP1* methylation elevates the level of the pseudogene sense transcript. In turn, a high level of this transcript correlates with a more favorable prognosis in endometrial cancer. The data obtained suggested that *PTENP1* methylation is associated with age-related changes in normal and hyperplastic endometrial tissues. We assumed that age-related increase in *PTENP1* methylation and subsequent elevation of its expression may serve as a protective mechanism aimed to prevent malignant transformation of endometrial tissue in women during the perimenopause, menopause, and postmenopause periods.

## Introduction

Methylation of cytosine at position 5 with the formation of 5-methylcytosine (5mC) is one of the most frequent epigenetic modifications of DNA in eukaryotes [1]. Normally, there are tissue-specific methylation patterns of the CpG sequences located at gene promoter regions, the so-called CpG islands (CGI). These patterns change in the course of ontogenesis and are often associated with suppression of transcription of relevant genes [2,3]. Considerable changes in DNA methylation occur over the life cycle of mammals [4,5]. Soon after fertilization, methylation level of genomic DNA in dividing blastomeres decreases considerably; then along with the fetus development it again reaches the initial values in all tissues except the primordial germ cells (PGCs). In the process of aging of an organism, total genomic DNA methylation level gradually decreases (a process known as global hypomethylation) while certain genetic loci are being selective hypermethylated. Analysis of patterns of such hypomethylation shows that accumulation of demethylated regions progresses linearly with age and mostly stochastically, apparently due to errors in the mechanism supporting the characteristic methylation patterns [6]. In contrast to this, age-related selective hypermethylation of DNA is more of a regular nature and depends on the tissue type [7]. The trend is the most pronounced in case of the sequences located in the promoter CGIs of the genes of transcription factors and specific receptors, reparation system genes, epigenetic regulation of genome functioning, and other genes, including those acting as tumor suppressors, as it has been demonstrated for human peripheral blood leukocytes [6,8]. The presence of the “programmed” and stochastic mechanisms involved in age-related changes of DNA methylation patterns is also supported by the studies in monozygotic twins [9]. Correlations observed between the level of methylation of individual genetic loci and chronological age of the organism triggered formation of an independent area of researches aimed to develop an epigenetic clock that would allow determining true biological age [10,11]. To predict the chronological age of an organism based on the analysis of its epigenetic markers, several specific sequences that are programmed to be hypo- or hypermethylated with aging are analyzed. Along with tissue-specific epigenetic markers, universal epigenetic markers have been discovered; the latter are DNA regions the methylation level of which changes synchronously with age in various organism tissues [12,13]. Since the signs of accelerated aging can be associated with many diseases, analysis of methylation of the above-mentioned sequences can be used for evaluation of the real biological age and prediction of pathology progression [14].

There is certain similarity in epigenetic changes occurring upon aging and in the course of cell malignization. In tumor transformation of tissues, similar to aging, global demethylation of cancer cell genome and locus-specific hypermethylation of individual regions of DNA was observed (for example, hypermethylation of tumor suppressor genes) [15], which partially resembles the state typical of normal embryonic stem cells. Most oncological diseases are characterized by a certain profile of differentially methylated regions of DNA [16].

Endometrial cancer is one of the most widely spread malignant tumors [17]. Changes in DNA methylation profiles associated with the pathological state have been actively studied. In endometrial cancer, hypermethylation affects tumor suppressor genes (for example, *MLH1*, *RASSF1A*, *APC*, *KLF4*, *ALDH1A2*, and *PCDH10*), as well as genes, the products of which are involved in cell adhesion (*SVEP1*), differentiation (*FGF12*, *TNFSF11*, *ASCL1*), and embryogenesis (*NODAL*, *TBX18*) [18–20]. In addition to protein-coding genes, hypermethylation in endometrial cancer was noted in the region of some enhancer sequences, as well as in a series of miRNA genes (*MIR25*, *MIR93*, *MIR99*, *MIR106B*, *MIR324*, *MIR3074*) and lncRNAs (e.g., *MEG3*) [19].

Gene of the PTEN tumor suppressor is often inactivated in endometrial cancer. *PTEN* expression is regulated by a transcribed pseudogene *PTENP1*. The pseudogene is located on chromosome 9p13.3 and is transcribed in two opposite directions to form long noncoding RNAs (lncRNAs): a sense transcript 3932 bp in length (PTENP1) and an antisense transcript (PTENP1-AS) of 888 bp. PTENP1-sence lncRNA competes with the *PTEN* gene mRNA for binding with inhibitory miRNAs and therefore increases PTEN protein level [21]. The antisense transcript of *PTENP1* pseudogene interacts with the promoter region of *PTEN* gene and inhibits its expression [22]. In our previous study we have not detected methylation of the *PTEN* minimal promoter region in endometrial cancer [23]. However, methylation of the 5'-terminal region of *PTENP1* was observed. Therefore, in this study we investigated in details methylation status of *PTENP1* in normal, hyperplastic and tumor tissues of endometrium obtained from women of various age groups. In addition, we analyzed the expression level of *PTENP1* and tested its possible connections with survival of the patients.

## Materials and methods

### Patients and tissue samples

Tissue samples were obtained from 236 patients of Blokhin Oncology Scientific Center and of Kulakov Research Center for Obstetrics, Gynecology, and Perinatology. All the patients included in our study were Caucasian women that lived in the same region (in Moscow) and did not suffer from severe concomitant diseases. Overall, 69 samples of normal endometrium (NE), mean age 39 ± 13.2 years; 64 samples of simple endometrial hyperplasia (EH), 47 ± 7.1 years; 45 samples of endometrial polyp (EP), 48 ± 9 years; and 58 samples of endometrioid carcinoma of endometrium, (EC, 62 ± 7.8 years) were analyzed. The tissues were collected during surgery or biopsy. Samples of NE tissues were collected from individuals who had not been diagnosed with EC, EH, or EP. Histology analysis was performed according to the criteria of the World Health Organization (WHO). Biological material was processed to the research laboratories after de-identification of the samples. The study was performed according to the principles proclaimed by Helsinki declaration. The study was approved by the ethics committees of the of Blokhin Oncology Scientific Center and Kulakov Research Center for Obstetrics, Gynecology, and Perinatology. All the patients gave written informed consent for their participation.

### Cell lines

In the study, we used 14 cell lines obtained from Koltsov Institute of Developmental Biology (Moscow, Russia), including xenograft epithelial keratinocytes (HaCaT), embrionic kidney (HEK-293), embryonic lung (MRC-5), pancreatic adenocarcinoma (AsPC-1, COLO-357, T3M4, BxPC-3), breast cancer (MCF-7, BT-474, HBL-100, and SKBR-3), cervical cancer (HeLa), ovarian cancer (SKOV-3), and a hepatocellular carcinoma (HepG2) cell lines. The HEK-293, HeLa, HepG2, MCF-7, MRC-5, SKBR-3, and SKOV-3 cells were cultured on the DMEM (PanEco, Russia) medium. The APC-1, BT-474, BxPC-3, COLO-357, HaCaT, HBL-100, and T3M-4 cells were cultured on the RPMI-1641 (PanEco, Russia) medium. Cultivation medium contained 10% (by volume) fetal embryonic serum, 4.5 g/L glucose, 2 mM L-glutamine, 100 IU/mL penicillin, and 100 μg/mL streptomycin. Cells were passaged 2–3 times per week till 80–90% monolayer was achieved. All the cells were incubated at 37°C under humidified atmosphere with 5% CO_2_.

### Genomic DNA and total RNA isolation and bisulfite modification of DNA

Genomic DNA from tissues and cells grown in culture was isolated by standard phenol–chloroform method [24]. Total RNA was isolated from cultured cells using the ExtractRNA kit (Evrogen, Russia) according the manufacturer’s protocol. RNA samples were treated with DNase I (Thermo Fisher Scientific, Lithuania) according to the manufacturer’s protocol. DNA and RNA concentrations were determined with the NanoDrop (Thermo Fisher Scientific, USA) spectrophotometer. DNA bisulfite modification of 1 μg of DNA was performed using the EpiTect Bisulfite Kit (Qiagen, Germany) according to the manufacturer’s recommendations.

### Global DNA demethylation

Cells were grown in a 25 cm^2^ culture flask up to 70% monolayer. Then, cells were incubated in the medium containing various concentrations of 5-Azacytidine (Serva, Germany). The concentration of 5-Azacytidine for each cell line was selected based on the results of cytotoxicity assay to obtain more than 80% survival rate: HEK-293–1.5 μM; HeLa—3 μM; HepG2–5 μM; MCF-7–26 μM; SKBR-3–35 μM; SKOV-3–7.5 μM and COLO-357–26 μM. The culture medium with 5-Azacytidine was changed every 24 h and after 72 h the treatment the cells were harvested. Glioblastoma PN19 cells were treated with 5-Aza-2’-deoxycytidine (Sigma-Aldrich, USA) at the final concentrations of 10 and 30 μM for 96 h. During this time, the culture medium with 5-Aza-2’-deoxycytidine was changed once after 48 hours.

### Methylation-specific PCR

Genomic DNA isolated from human blood lymphocytes, methylated in vitro by the SssI methyltransferase (SibEnzyme, Russia) and treated with sodium bisulfite was used as methylated control. Bisulfite-converted DNA from blood lymphocytes was used as non-methylated control. PCR mixture (25 μL) contained 0.5 μM forward and reverse primers, 0.5–1 units of Taq DNA polymerase, and 25 ng bisulfite-converted DNA. The reaction was performed under the following conditions: primary denaturation at 95°C for 5 min; 33 cycles of denaturation at 95°C for 30 s, annealing at 63–64°C for 30 s, elongation at 72°C for 30 s; and final elongation at 72°C for 3 min. The sequences of MS-PCR primers [25] and PCR-product sizes are shown in S1 Table. PCR products (5 μL) were analyzed using ethidium bromide-stained agarose gel (3%) electrophoresis. We defined our samples as “methylated” if we obtained the PCR product with the primers for the methylated template. We defined the sample as “unmethylated” if we had only the band for the unmethylated DNA. The original uncropped and unadjusted images of all gels are shown in S1 Fig.

### Sanger DNA sequencing

MS-PCR products were purified using QIA quick Gel Extraction Kit (Qiagen, Germany), in accordance with the manufacturer's protocol. Sequencing of the PCR products was carried out by the Sanger method using a Thermo Sequenase Cycle Sequencing Kit (Amersham Bio Sciences, USA). Electrophoretic separation of the products was performed using an automatic ALF Express II sequencer (Amersham Bio Sciences, USA).

### Cytotoxicity assay

To study 5-Azacytidine cytotoxicity cells were seeded onto 96-well plates (3 × 10^3^ cells per well) and incubated overnight. Then, the medium was replaced with a fresh one containing 0.5, 1, 2, 3–10, 20, 30–90 or 100 μM 5-Azacytidine. Cells were incubated at 37˚C in the atmosphere of 5% CO_2_ for 72 h. The medium with 5-Azacytidine was changed every 24 h. Cell viability was evaluated using MTT assay [26]. The optical density was measured on a StatFax-2100 Microplate reader (Awareness Technology, USA) at a wavelength of λ = 545 nm. An example of a cell survival curve (for HeLa cells) is shown in S2 Fig.

### qRT-PCR

cDNA was synthesized using MMLV RT kit (Evrogen, Russia) according the manufactures’s recommendations. SYBR Green RT-qPCR method was used to evaluate the RNA levels in each sample. Amplification was carried out on an ANK-37 (DNA-technology, Russia). β-actin and 18S were used as the internal control genes. The relative RNA expression level was calculated using the 2^−ΔΔCt^ method [27]. The sequences of the primers and the conditions of PCR-amplification are shown in S1 Table.

### Bioinformatic analysis

Relationship between *PTENP1* methylation and overall survival of the patients was determined using the MethSurv software [28]. Relationship between *PTENP1* expression and overall survival of the patients was determined using the OncoLnc software [29]. To study PTENP1 and PTENP1-AS expression in healthy patients of different age we used previously published RNAseq data (GSE102131) [30]. Reads were quantified against Homo Sapiens GRCh38.13 genome annotation genome annotation at the transcript level using Salmon. Results were aggregated to gene level using tximport. In brief, the datasets were filtered to remove rows with only a single count across all samples and differentially expressed genes were identified using DESeq2.

### Statistic analysis

All data are presented as mean ± SD. Each experiment was performed in three replicates. Statistical differences between two groups were evaluated by two tailed t-test. One-way ANOVA was utilized in comparisons of more than 2 groups, following Dunnett’s/Tukey’s posttest. The statistical significance of Kaplan–Meier survival plot was determined by log-rank analysis. Statistical analysis was performed by Prism 6 (Graphpad Software). P < 0.05 was considered statistically significant.

## Results

### Methylation of the *PTENP1* pseudogene in human cell lines

**Fig 1:**
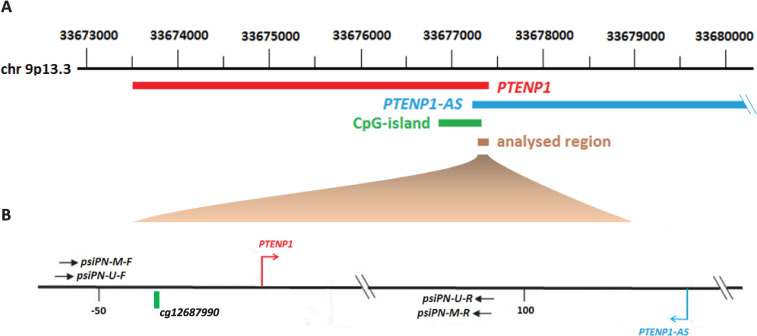
*PTENP1* pseudogene sequence (A) and the analyzed region of the pseudogene (B). Red and blue arrows indicate the sites of transcription initiation of the sense (PTENP1) and antisense (PTENP1-AS) transcripts of *PTENP1* gene; black arrows indicate the location of primers for MS-PCR; *cg12687990—*the probe from llumina Infinium Human Methylation 450 (HM450K) bead array, which was used to determine *PTENP1* methylations level in samples from TCGA database.

We first used methylsensitive PCR (MS-PCR) to evaluated the methylation status of the CpG island located at *PTENP1* 5'-termial region in human cell lines. This region includes the transcription start site of *PTENP1-*sence long non-coding RNA and contains 18 CpG-pairs (Fig 1) while the primer for MS-PCR annealed to 5 CpG sites.

**Fig 2:**
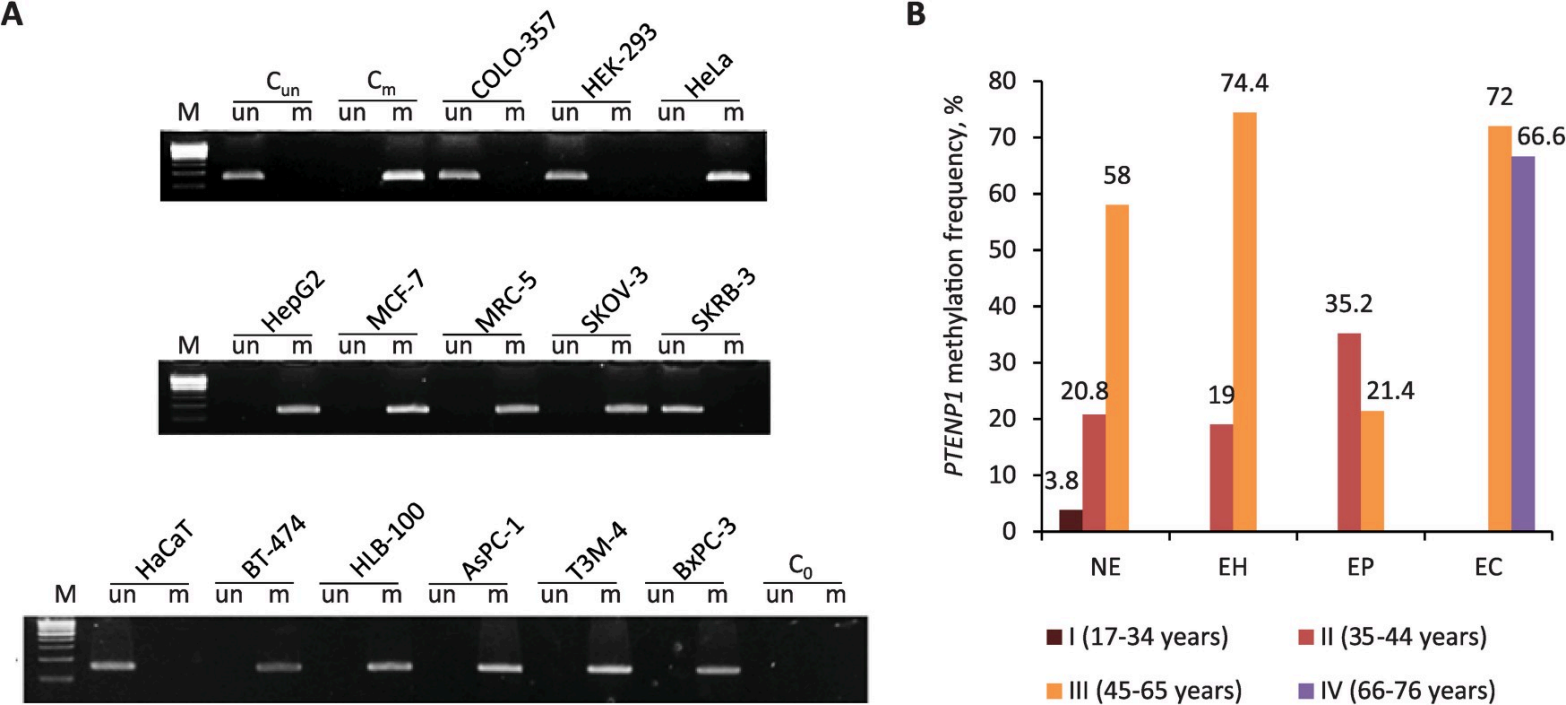
*PTENP1* pseudogene methylation in cell lines (A) and malignant and non-malignant endometrial tissues (B). C_un_, unmethylated control (DNA obtained from peripheral blood and treated with sodium bisulfite), C_m_, methylated control (DNA obtained from peripheral blood, methylated with SssI methyltransferase, and treated with sodium bisulfite), C_0_, amplification without a DNA template; un and m–PCR amplification with primers for unmethylated and methylated DNA respectively; M–a molecular weight marker; NE, normal endometrium; EH, endometrial hyperplasia; EP, endometrial polyps; EC, endometrial carcinoma.

In total we studied 14 conventional cell lines, three of which (HaCaT, HEK-293, MCR-5) were noncancerous and the remaining ones were related to pancreatic adenocarcinoma (AsPC-1, COLO-357, T3M4, BxPC-3), breast cancer (MCF-7, BT-474, HBL-100, and SKBR-3), cervical cancer (HeLa), ovarian cancer (SKOV-3), and a hepatocellular carcinoma (HepG2). The pseudogene was found to be unmethylated in 2 out of 3 noncancerous cell lines (HaCaT and HEK-293) and methylated in 9 out of 11 cell lines obtained from malignant tissues (AsPC-1, BT-454, BxPC, HBL-100, HeLa, HepG2, MCF-7, SKOV-3, and T3M-4). Remaining 2 cancer cell lines that had unmethylated region of the *PTENP1* were obtained from pancreatic adenocarcinoma and breast cancer (COLO-357 and SKBR-3 respectively) (Fig 2A).

### Methylation of *PTENP1* in samples of malignant and non-malignant endometrium

Next, we studied methylation status of *PTENP1* in 236 clinical samples of malignant and non-malignant endometrium. The MS-PCR results for 13 are shown in S3 Fig and were confirmed by Sanger sequencing of the obtained PCR fragments (S4 Fig).

According to our data, we did not find significant differences in the frequency of pseudogene methylation between samples of normal endometrium and endometrial polyps (24% and 26.6% respectively). At the same time, we found such a difference between samples of normal endometrium and endometrial hyperplasia (24% and 56.2%, respectively) and normal endometrium and endometrial carcinoma (24% and 68.9%, respectively). However, these patient groups differed significantly in their mean age, which made such a comparison inaccurate. Therefore, we decided to divide the patients into age groups and compare the frequencies of *PTENP1* methylation in normal endometrium, endometrial polyps, endometrial hyperplasia and endometrial carcinoma within each of the age groups, as well as compare the age groups with each other. We included in our study age group I (17–34 years old), consisting of women of reproductive age, a group of women of late reproductive age (35–44 years old, age group II), a group of perimenopausal, menopausal and postmenopausal women (45–65 years old, age group III). Among the patients suffering from EC, there were women 66–76 years old (age group IV). Average age for each group, number of patients and endometrium tissue types are indicated in Table 1.

**Table 1:** Patients and tissue samples.

Age groups	Number of patients; mean age ± sd			
	NE	EH	EP	EC
group I 17–34 years	n = 26; 23.5±5.0	–	–	–
group II 35–44 years	n = 24; 40.0±2.9	n = 21; 40.0±2.6	n = 17; 39.0±2.9	–
group III 45–65 years	n = 19; 52.5±6.0	n = 43; 52.1±6.5	n = 28; 55.0±6.3	n = 25; 55.0±3.3
group IV 66–76 years	–	–	–	n = 33; 70.0±3.2

Comparison of the *PTENP1* methylation frequencies in various endometrium tissue types (NE, EH, and EP) in women of age group II did not reveal any significant differences. The same applies to age group III, excluding the EP subgroup (Table 2, Fig 2B). *PTENP1* methylation frequency in the EP patients of group III was considerably lower than in women of the same age group with NE, EH, or EC (p < 0.05).

**Table 2:** Significance of differences in the *PTENP1* methylation frequency in II and III age groups between various types of endometrial tissue.

Tissue type	NE	EH	EP	EC
Age group II (35–44 years)				
NE	-	*p* = 1.000	*p* = 0.467	-
EH	*p* = 1.000	-	*p* = 0.293	-
EP	*p* = 0.467	*p* = 0.293	-	-
Age group III (45–65 years)				
NE	-	*p* = 0.748	*p* = 0.015	*p* = 0.375
EH	*p* = 0.748	-	*p* = 0.014	*p* = 0.766
EP	*p* = 0.015	*p* = 0.014	-	*p* = 0.000
EC	*p* = 0.375	*p* = 0.766	*p* = 0.000	-

Next we compared different age groups with each other. In the case of normal endometrium *PTENP1* methylation was detected in 3.8% of samples obtained from women of age group I, in 20.8% of samples of age group II, and in 58% of samples of age group III (Table 3, Fig 2B). Significant differences in methylation frequency of the *PTENP1* pseudogene were found between age groups I and III, as well as II and III (p < 0.05). Thus, *PTENP1* methylation in normal endometrium starts to be particularly pronounced in women aged 45 years or older, which can be associated with approaching or ongoing menopause.

**Table 3:** *PTENP1* methylation analysis in endometrial tissues of women of various age groups.

Age group of patients	Tissue samples with methylated *PTENP1*	% of samples with methylated *PTENP1*	*p*-value of differences
Normal endometrium			
I (17–34 years), n = 26	1	3.8	0.093 (I vs II)
II (35–44 years), n = 24	5	20.8	0.000 (I vs III)
III (45–65 years), n = 19	11	58.0	0.025 (II vs III)
Endometrial hyperplasia			
II (35–44 years), n = 21	4	19	0.016
III (45–65 years), n = 43	32	74.4	
Endometrial polyps			
II (35–44 years) n = 17	6	35.2	0.325
III (45–65 years), n = 28	6	21.4	
Endometrial carcinoma			
III (45–65 years), n = 25	18	72.0	0.778
IV (66–76 years), n = 33	22	66.6	

The same trend was observed among the patients with endometrial hyperplasia. Here, the difference between II and III age groups was also significant (19 and 74.4% respectively). At the same time, we did not find significant differences in the frequencies of pseudogene methylation between the age groups of patients with endometrial carcinoma and women with endometrial polyps.

To confirm our data, we next divided the patients into new set of age groups with the age interval of 10–11 years: 1 (17–24 years), 2 (25–34 years), 3 (35–44 years), 4 (45–54 years), 5 (55–65 years) and 6 (66–76 years) (S2 Table and S5A Fig). We did not find statistically significant differences between groups 1–3, as well as between groups 4 and 5 of women with normal endometrium (S3 Table). At the same time, a comparison of NE tissue samples from women under 45 years with groups of women over 45 years old revealed statistically significant differences in the frequencies of *PTENP1* methylation. The difference in methylation frequency between groups 3 (35–44 years) and 4 (45–54 years) was close to statistically significant (*p* = 0.067). For endometrial hyperplasia we also observed significant differences between patients under and over 45 years old (S4 Table). For endometrial polyps the differences between age groups of patients were not significant (S5 Table). Also there were no statistically significant differences between age groups of patients with endometrial carcinoma (S6 Table).

### *PTENP1* expression in normal endometrium obtained from women of different age groups

**Fig 3:**
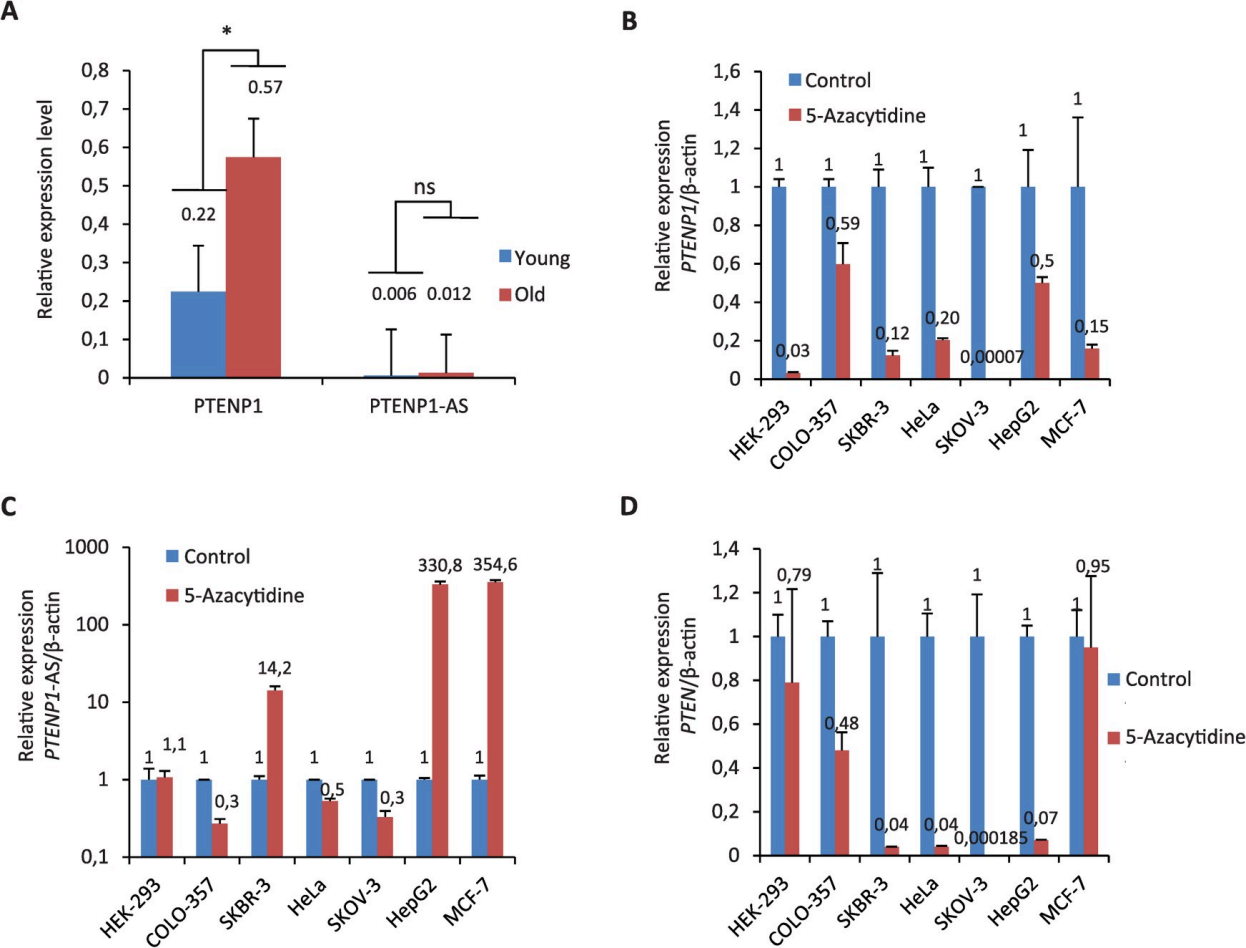
The mean relative levels of PTENP1 and PTENP1-AS transcription in NE tissue from women of two age groups (A), the relative levels of PTENP1 (B), PTENP1-AS (C) and PTEN (D) transcripts in human cells, treated with 5-Azacytidine. Group “Young”: 23–30 years (mean age 29±2.07 years, n = 10); group “Old”: 40–43 years (mean age 41.5±1.34 years, n = 10); * *p* = 0.0313; ns–not significant (*p* = 0,739); for each cell line untreated cells were used as controls (arbitrary set at 1).

Since we found an age-dependent increase in the frequency of methylation of *PTENP1* in normal and hyperplastic endometrium, we suggested that the level of expression of the pseudogene should also change with age in these tissues. To confirm our hypothesis, we carried out a bioinformatic analysis of previously published RNA sequencing data obtained from normal endometrium of 10 women 23–30 years old (group “Young”) and of 10 women 40–43 years old (group “Old”) [30]. We evaluated the expression levels of two long non-coding RNAs that are known to be expressed from *PTENP1* pseudogene: PTENP1 and PTENP1-AS. The level of PTENP1-AS was very low in both groups and therefore we did not find statistically significant difference in its expression (Fig 3A). On the other hand, the level of PTENP1 was significantly different in these two groups (p<0.05). Surprisingly, PTENP1expression was almost 3 folds higher in older women as compared to younger ones. Thus, we revealed an age-dependent increase in the level of PTENP1 expression in healthy endometrium.

### Effects of *PTENP1* methylation on the level of PTENP1, PTENP1-AS and PTEN in human cell lines

In the experiments described above we have demonstrated that *PTENP1* methylation is increased with age of the patients and that expression of PTENP1 RNA is also upregulated in older women. Therefore, we can propose that methylation of *PTENP1* may promote its’ expression. To further study the relationship between DNA methylation and *PTENP1* expression we have tested the effect of 5-Azacytidine (CpG hypomethylating agent) on the expression of PTENP1, PTENP1-AS and PTEN transcripts. Analysis of 7 human cell lines (COLO-357, HEK-293, HeLa, HepG2, MCF-7, SKBR-3 and SKOV-3) treated with different concentrations of 5-Azacytidine revealed that inhibition of DNA methylation significantly reduces PTENP1 level in all tested cell lines. At the same time the level of PTENP1-AS was unchanged in HEK-293 cells and increased in SKBR-3, HepG2 and MCF-7 cells (Fig 3C). The level of *PTEN* gene expression was decreased in most cells treated with 5-Azacytidine with the exception of HEK-293 and MCF-7 cells (the level remained unchanged). Interestingly, the result that 5-Azacytidine downregulates *PTENP1* expression contradicts to the previously published observation that 5-Aza-2′-deoxycytidine upregulates *PTENP1* expression in 4 out of 5 renal cell lines [31]. Therefore, we performed additional experiments using 5-Aza-2′-deoxycytidine. According to our data 5-Aza-2′-deoxycytidine similarly to 5-Azacytidine was able to downregulate *PTENP1* expression and upregulate PTENP1-AS transcript level in a concentration dependent manner (S5B Fig). Altogether, our data confirm that in all 8 tested cell lines CpG methylation may promote *PTENP1* expression.

### Effect of *PTENP1* expression and methylation on the overall survival of cancer patients

**Fig 4:**
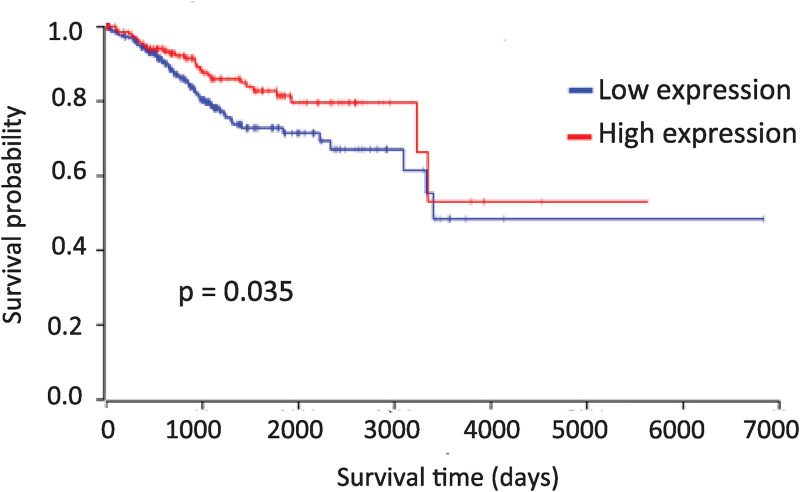
The Kaplan-Meier survival curve for patients with EC divided into two groups based on the *PTENP1* expression level. The number of patients in each group and the *p* value (log-rank test) are indicated.

The sense transcript of *PTENP1* pseudogene exhibits the properties of tumor suppressive lncRNA in many types of human cancer [31–33]. Our results, presented above, showed that the level of this transcript in normal endometrium increases significantly after 40 years. We hypothesized that PTENP1 sense non-coding RNA may play a protective role by decreasing aggressiveness of endometrial carcinoma which usually develops in women over 40. In order to test this hypothesis, we investigated the relationship between the level of *PTENP1* expression and the overall survival of patients with endometrial carcinoma. We analyzed data from 540 endometrial carcinoma patients and found that an increased level of pseudogene expression indeed correlates with the prolonged overall survival (Fig 4).

**Fig 5:**
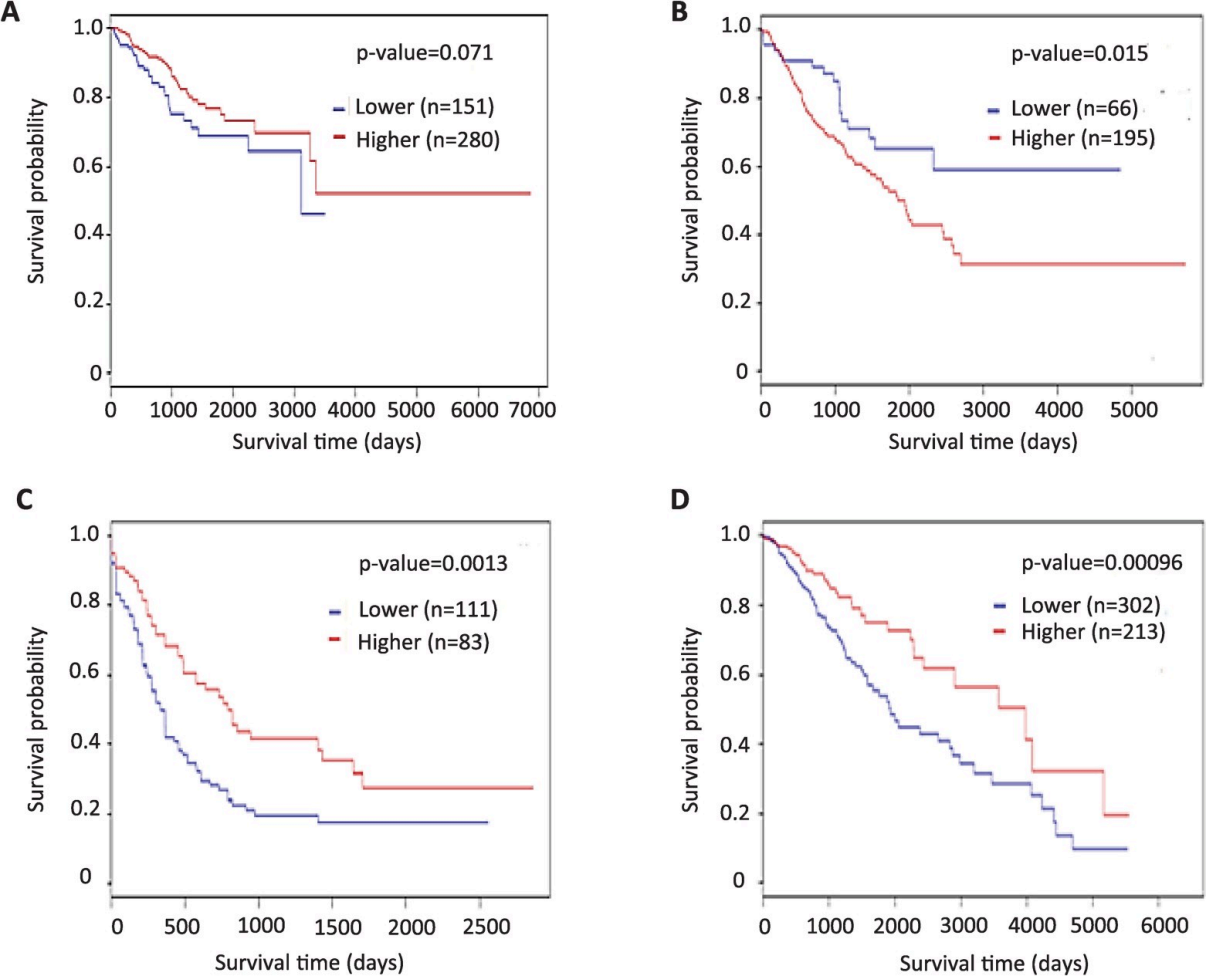
The Kaplan-Meier survival curve for patients with endometrial cancer (A), sarcoma (B), acute myeloid leukemia (C) and lower grade glioma (D) divided into two groups based on the *PTENP1* methylation level. The number of patients in each group and the *p*-value (log-rank test) are indicated.

According to our data methylation of the pseudogene may positively regulate the transcription of its sense noncoding RNA. Therefore, next, we tested the relationship between patients’ survival and the methylation level of CpG island in the promoter region of *PTENP1*. We analyzed the TCGA methylome data obtained from 431 patients with endometrial carcinoma, 261 patients with sarcoma, 194 patients with acute myeloid leukemia and 515 patients suffering from lower grade glioma. As shown in Fig 5A, for endometrial carcinoma we found some correlation between higher *PTENP1* methylation and more favorable prognosis for patients, however the differences were not quite statistically significant (p = 0.071). On the other hand, in acute myeloid leukemia and lower grade glioma patients *PTENP1* hypermethylation strongly correlated with favorable prognosis (Fig 5C and 5D). In contrast, in sarcoma, hypermethylation of pseudogene was found in patients with shorter survival (Fig 5B). These data may indicate the tissue-specific effect of PTENP1 on the phenotype of cancer cells.

## Discussion

Data obtained in this study demonstrates that *PTENP1* methylation may reflect age-related changes that occur in endometrium after the age of 45 and may indirectly indicate the approach or onset of menopause. This statement may be true for normal endometrium, endometrial hyperplasia, and endometrial carcinoma, but not for endometrial polyps, since the frequency of pseudogene methylation in EP tissue does not change with age. EPs belong to the group of benign neoplasms rising due to excess proliferation of epithelium glands and stroma [34]. They appear in both women of reproductive age and those at menopausal or post-menopausal stage. Of note, according to the literature, EPs have a number of histological features distinguishing it from normal endometrial tissue and from EH. For example, this neoplasm is characterized by certain positioning and structural abnormalities of glands. There are data demonstrating that EP consists of immature endometrium cells that are not subjected to cyclic changes in contrast to NE and EH [34]. In this study we showed that EP has also epigenetic peculiarities: the frequency of *PTENP1* methylation in EP does not depend on patient’s age.

It was previously shown that patterns of DNA methylation in normal endometrium are not constant. The epigenetic clock test based on methylation patterns of 353 CpG sites, developed by Horvath and co-workers [35], demonstrated a correlation between chronological age of a woman at menopause and biological (reproductive) age determined using the DNA from blood cells [36]. In menopause, biological aging was observed to accelerate according to the epigenetic clock. We do not currently have enough data to assert that *PTENP1* region analyzed in our study belongs to epigenetic clock-sites and that it can be used for accurate calculating the biological age. However, we believe that the trend for increased methylation of the *PTENP1* pseudogene in older patients could be related to the approaching or ongoing menopause.

Since we identified an age-dependent increase in the level of PTENP1 and an increase in the frequency of pseudogene methylation in the normal endometrium, we suggested that methylation of *PTENP1* may be a positive regulator of the expression of this pseudogene. Incubation of cultured cells with methylase inhibitors 5-Azacytidine or 5-Aza-2’-deoxycytidine led to a decrease in the PTENP1 expression in all 8 tested cell lines. At the same time, *PTEN* gene was also downregulated in 6 cell lines. These results suggested that *PTENP1* methylation contributes to an increase in the level of PTENP1 sense non-coding RNA as well as PTEN mRNA. Interestingly, the decrease in the level of PTENP1 after treatment with 5-Azacytidine was found not only in those lines in which we detected methylation of the pseudogene promoter (HeLa, SKOV-3, HepG2 and MCF-7), but also in those lines in which we did not detect the methylation (HEK-293, COLO-357 and SKBR-3). However, it should be noted that we analyzed only a relatively small fragment of the *PTENP1* CpG island and the absence of methylation in this region does not exclude this modification in other regions of the CpG island sequence.

The sense transcript of *PTENP1* is a positive regulator of *PTEN* gene expression [21]. It acts as a lncRNA that competes with PTEN mRNA for the binding of inhibitory miRNAs. In this context, it is not surprising that a decrease in the level of sense RNA leads to a decrease in the level of mRNA of the *PTEN* gene. It is more intriguingly that the treatment with the methylase inhibitors resulted in a decrease in the level of the pseudogene sense transcript. As a general rule, methylation of gene promoter regions leads to a decrease in the level of gene expression [37]. However, there are evidences that methylation can have the opposite effect. Thus, hypermethylation of 5’-noncoding region (from -441 to -218) of *hTERT* gene is observed in all of the cell lines possessing telomerase activity, while the region is demethylated in all of telomerase-negative samples [38]. In addition, methylation of exons 1 and 2 also increases the level of *hTERT* gene expression. It was shown that these exons include binding sites for the transcriptional repressor CTCF. When the sequences are methylated, the CTCF binding efficiency is very low. In the absence of methylation, CTCF binds to the gene and suppress its transcription [39]. Another example is methylation of the Survivin gene promoter. The promoter methylation could inhibit the binding of p53 (a repressor of Survivin expression) and leads to an increase in mRNA level [40]. Therefore, it is likely that *PTENP1* belongs to the group of genes which expression is positively regulated by DNA methylation.

Since *PTENP1* methylation is likely to contribute to an increase in its expression level, it can be assumed that this epigenetic modification should correlate with a more favorable prognosis for patients with cancer. Indeed, according to our data analysis, in lower grade glioma, acute myeloid leukemia and endometrial cancer patients with higher methylated *PTENP1* show prolonged survival. In contrast, in sarcoma, *PTENP1* methylation is correlated with a poor prognosis. These discrepancies may be due to the fact that the functions of PTENP1 may differ in various tissues. PTENP1 sequence contains binding sites for many miRNAs, and therefore it can participate in the regulation of multiple genes in addition to oncosuppressor *PTEN*. For example, Chengping Wu and colleagues showed that PTENP1 may serve as a sponge for miR-27a-3p to upregulate EGR1 level in cervical cancer cells. As a result, PTENP1 suppress cell growth, motility and epithelial-to-mesenchymal transition and inhibits cervical cancer progression [41]. On the other hand, Yndestad S. et al. demonstrated that PTENP1 may play a prooncogenic role and can enhance proliferation of ER-positive breast cancer cells MSF-7 and accelerate growth of corresponding tumors in vivo by downregulating ER-α protein level [42]. These observations may explain opposite role of PTENP1 in different types of malignancies.

In our previous study we demonstrated the absence of a correlation of *PTENP1* methylation with the clinical characteristics of endometrial cancer (disease stage, FIGO grade, myometrium invasion) [23]. Here, we found higher frequency of *PTENP1* methylation in patients over 45 years with EH and EC, which did not differ from methylation of NE in women of matched age groups. The data obtained indicated that *PTENP1* methylation is associated with age-related changes, but not with malignancy of endometrial tissue.

## Conclusion

Our data allow us to conclude that *PTENP1* methylation reflects age-related changes that occur in normal and hyperplastic endometrium (but not in endometrial polyps). We also demonstrated an age-dependent increase in the level of pseudogene expression, which in turn correlates with a better prognosis of endometrial carcinoma. The obtained data suggest that age-related increase in the frequency of *PTENP1* methylation and elevation in the level of its’ expression may serve as a protective mechanism aimed to prevent malignant transformation of endometrial tissue in women during the perimenopause, menopause, and postmenopause periods. We hope that our study will expand current understanding of the role of pseudogenes and their transcripts in physiological processes, including the aging of the human body.

## Supporting information

S1 Fig The raw images. (TIF)

S2 Fig Viability of HeLa cells after treatment with 5-Azacytidine. (TIF)

S3 Fig Analysis of *PTENP1* methylation by MS-PCR in women with NE (A), patients with EH (B), EP (C) and EC (D). 1, 2, 3 **–**number of DNA samples; C_un_, unmethylated control (DNA obtained from peripheral blood and treated with sodium bisulfite), C_m_, methylated control (DNA obtained from peripheral blood, methylated with SssI methyltransferase, and treated with sodium bisulfite), C_0_, amplification without a DNA template; un and m–PCR amplification with primers for unmethylated and methylated DNA respectively; M–a molecular weight marker.
(TIF)

S4 Fig DNA sequencing of PCR products, obtained from unmethylated (the top sequence), partly methylated (two middle sequences) and methylated templates (two bottom sequences). The top row of letters means the sequence of *PTENP1* from the Gene Bank; the bottom row–*PTENP1* sequence obtained as a result of DNA sequencing. The dotted line denotes cytosine residues that were converted in unmethylated DNA samples or remained unconverted in methylated DNA samples.
(TIF)

S5 Fig *PTENP1* methylation status in different age groups of women with NE, EH, EP and EC (A); the relative levels of PTENP1 and PTENP1-AS transcripts in human glioblastoma cells, treated with 5-Aza-2′-deoxycytidine (B). DMSO treated cells were used as controls.
(TIF)

S1 Table PCR primers and amplification conditions. Ta–the temperature of PCR-primer’s annealing.
(DOC)

S2 Table The patients age groups including the age range of 10–11 years. (DOC)

S3 Table Comparison of *PTENP1* methylation frequency in different age groups of women with NE. (DOC)

S4 Table Comparison of *PTENP1* methylation frequency in different age groups of patients with EH. (DOC)

S5 Table Comparison of *PTENP1* methylation frequency in different age groups of patients with EP. (DOC)

S6 Table Comparison of *PTENP1* methylation frequency in different age groups of patients with EC. (DOC)

## References

[1] Schmitz RJ, Lewis ZA, Goll MG. DNA methylation: shared and divergent features across eukaryote. Trends Genet.2019 11; 35(11): 818–27. 10.1016/j.tig.2019.07.007 31399242 PMC6825889

[2] Deaton AM, Bird A. CpG islands and the regulation of transcription.Genes Dev. 2011 5; 25(10): 1010–22. 10.1101/gad.2037511 21576262 PMC3093116

[3] Ziller MJ, Gu H, Muller F, Donaghey J, Tsai LTY, Kohlbacher O, et al Charting a dynamic DNA methylation landscape of the human genome. Nature 2013 8; 500 (7463): 477–81. 10.1038/nature12433 23925113 PMC3821869

[4] Hackett JA, Surani MA. DNA methylation dynamics during the mammalian life cycle. Philos Trans R SocLond B Biol Sci. 2013 1; 368(1609): 20110328 10.1098/rstb.2011.0328 23166392 PMC3539357

[5] Luo C, Hajkova P, Ecker JR. Dynamic DNA methylation: in theright place at the right time. Science Sep; 361(6409): 1336–40. 10.1126/science.aat6806 30262495 PMC6197482

[6] Johansson A, Enroth S, Gyllensten U. Continuous aging of the human DNA methylome throughout the human lifespan. PLoS One 2013 6; 8(6): e67378 10.1371/journal.pone.0067378 23826282 PMC3695075

[7] Christensen BC, Houseman EA, Marsit CJ, Zheng S, Wrensch MR, Wiemels JL, et al Aging and environmental exposures alter tissue-specific DNA methylation dependent upon CpG island context. PLoS Genet. 2009 8; 5 (8): e1000602 10.1371/journal.pgen.1000602 Epub 2009 Aug 14; 19680444 PMC2718614

[8] Rakyan VK, Down TA, Maslau S, Andrew T, Yang T-P, Beyan H, et al Human aging-associated DNA hypermethylation occurs preferentially at bivalent chromatin domains. Genome Res. 2010 4; 20(4): 434–9. 10.1101/gr.103101.109 20219945 PMC2847746

[9] Fraga MF, Ballestar E, Paz MF, Ropero S, Setien F, Ballestar ML, et al Epigenetic differences arise during the lifetime of monozygotic twins. Proc Natl AcadSci U S A 2005 7; 102(30): 10604–9. 10.1073/pnas.0500398102 16009939 PMC1174919

[10] Field AE, Robertson NA, Wang T, Havas A, Ideker T, Adams PD. DNA methylation clocks in aging: categories, causes, and consequences. Mol Cell. 2018 9; 71(6): 882–95. 10.1016/j.molcel.2018.08.008 30241605 PMC6520108

[11] Bell CG, Lowe R, Adams PD, Baccarelli AA, Beck S, Bell JT, et al DNA methylation aging clocks: challenges and recommendations. Genome Biol. 2019 11; 20(1): 249 10.1186/s13059-019-1824-y 31767039 PMC6876109

[12] Jones MJ, Goodman SJ, Kobor MS. DNA methylation and healthy human aging. Aging Cell. 2015 12; 14(6): 924–32. 10.1111/acel.12349 25913071 PMC4693469

[13] Horvath S, Raj K. DNA methylation-based biomarkers and the epigenetic clock theory of ageing. Nat. Rev. Genet. 2018 6; 19(6): 371–384. 10.1038/s41576-018-0004-3 . 29643443

[14] Ashapkin VV, Kutueva LI, Vanyushin BF. Epigenetic Clock: Just a Convenient Marker or an Active Driver of Aging? Adv Exp Med Biol. 2019; 1178: 175–206. 10.1007/978-3-030-25650-0_10 . 31493228

[15] Zhang H, Zhang K, Zhu J. A model for aberrant DNA methylomes in aging cells and cancer cells. BiochemSoc Trans. 2019 8; 47(4): 997–1003. 10.1042/BST20180218 . 31320500

[16] Sproul D, Kitchen RR, Nestor CE, Dixon JM, Sims AH, Harrison DJ, et al Tissue of origin determines cancer-associated CpG island promoter hypermethylation patterns. Genome Biol. 2012 10; 13(10): R84 10.1186/gb-2012-13-10-r84 23034185 PMC3491412

[17] Murali R., Soslow RA, Weigelt B. Classification of endometrial carcinoma: more than two types. Lancet Oncol. 2014 6; 15(7): e268–78. 10.1016/S1470-2045(13)70591-6 . 24872110

[18] Pallares J, Velasco A, Eritja N, Santacana N, Dolcet X, Guatrecasas M, et al Promoter hypermethylation and reduced expression of *RASSF1A* are frequent molecular alterations of endometrial carcinoma. Mod Pathol. 2008 6; 21(6): 691–9. 10.1038/modpathol.2008.38 . 18469797

[19] Zhang B, Xing XY, Li J, Lowdon R, Zhou Y, Lin N, et al Comparative DNA methylome analysis of endometrial carcinoma reveals complex and distinct deregulation of cancer promoters and enhancers. BMC Genomics. 2014 10; 15: 868 10.1186/1471-2164-15-868 25286960 PMC4198682

[20] Trimarchi MP, Yan P, Groden G, Bundschuh R, Godfellow PJ. Identification of endometrial cancer methylation features using combined methylation analysis methods. PLoS ONE. 2017 3; 12 (3), e0173242, 10.1371/journal.pone.0173242 28278225 PMC5344376

[21] Poliseno L, Salmena L, Zhang J, Carver B, Haveman WJ, Pandolfi PP. A coding-independent function of gene and pseudogene mRNAs regulates tumor biology. Nature 2010 6; 465(7301): 1033–8. 10.1038/nature09144 20577206 PMC3206313

[22] Johnsson P, Ackley A, Vidarsdottir L, Lui W, Corcoran M, Grander D, et al A pseudogene long-noncoding-RNA network regulates *PTEN* transcription and translation in human cells. Nat StructMol Biol. 2013 4; 20(4): 440–6. 10.1038/nsmb.2516 23435381 PMC3618526

[23] Kovalenko TF, Morozova KV, Ozolinya LA, Lapina IA, Patrushev LI. The *PTENP1*pseudogene, unlike the *PTEN* gene, is methylated in normal endometrium, as well as in endometrial hyperplasias and carcinomas in middle-aged and elderly females. ActaNaturae. 2018 Jan-Mar; 10(1): 43–50. 29713518 PMC5916733

[24] Lindblom B, Holmlund G. Rapid DNA purification for restriction fragment length polymorphism analysis. Gene Anal Tech. 1988 Sep-Oct; 5(5): 97–101. 10.1016/0735-0651(88)90003-9 . 2903845

[25] Marsit CJ, Zheng S, Aldape K, Hinds PW, Nelson HH, Wiencke JK, et al *PTEN* expression in non-small-cell lung cancer: evaluating its relation to tumor characteristics, allelic loss, and epigenetic alteration. Hum Pathol. 2005 7; 36(7): 768–76. 10.1016/j.humpath.2005.05.006 . 16084946

[26] Mosmann T. Rapid colorimetric assay for cellular growth and survival: application to proliferation and cytotoxicity assays. J Immunol Methods. 1983 12; 65(1–2): 55–63. 10.1016/0022-1759(83)90303-4 . 6606682

[27] Livak K, Schmittgen TD. Analysis of relative gene expression data using real- time quantitative PCR and the 2(-Delta Delta C(T)) Method. Methods. 2001 12; 25(4): 402–8. 10.1006/meth.2001.1262 . 11846609

[28] Modhukur V, Iljasenko T, Metsalu T, Lokk K, Laisk-Podar T, Vilo J. MethSurv: a web tool to perform multivariable survival analysis using DNA methylation data. Epigenomics. 2018 3; 10(3): 277–288. 10.2217/epi-2017-0118 . 29264942

[29] Anaya J. OncoLnc: linking TCGA survival data to mRNAs, miRNAs, and lncRNAs. PeerJ Computer Science 2016; 2:e67 10.7717/peerj-cs.67.

[30] Brighton P, Yojiro M, Fishwick K, Vrljicak P, Tewary S, Fujihara R, et al Genome wide expression analysis of young and aged human endometrium; 2020 [cited 2020 Jun 08]. Available from: https://www.ncbi.nlm.nih.gov/geo/query/acc.cgi?acc=GSE102131.

[31] Yu G, Yao W, Gumireddy K, Li A, Wang J, Xiao W, et al Pseudogene PTENP1 functions as a competing endogenous RNA to suppress clear-cell renal cell carcinoma progression. Mol Cancer Ther. 2014 12; 13(12): 3086–3097. 10.1158/1535-7163.MCT-14-0245 . 25249556 PMC4265235

[32] Qian Y, Li K, Liu Q and Liu Z. Long non-coding RNA PTENP1 interacts with miR-193a-3p to suppress cell migration and invasion through the PTEN pathway in hepatocellular carcinoma. Oncotarget. 2017 11; 8(64): 107859–869. 10.18632/oncotarget.22305 . 29296207 PMC5746109

[33] Hu S, Xu L, Li L, Luo D, Zhao H, Li D, et al Overexpression of lncRNA PTENP1 suppresses glioma cell proliferation and metastasis in vitro. Onco Targets Ther. 2018 12; 12: 147–156. 10.2147/OTT.S182537 . 30613153 PMC6306071

[34] Nijkang NP, Anderson L, Markham R, Manconi F. Endometrial polyps: pathogenesis, sequelae and treatment. SAGE Open Med. 2019 May; 7: 2050312119848247 10.1177/2050312119848247 eCollection 2019. 31105939 PMC6501471

[35] Horvath S. DNA methylation age of human tissues and cell types. Genome Biol. 2013 14(10): R115 10.1186/gb-2013-14-10-r115 24138928 PMC4015143

[36] Levine ME, Lua AT, Chen BH, Hernandez DG, Singleton AB, Ferruccic L, et al Menopause accelerates biological aging. PNAS 2016 8; 113(33): 9327–32. 10.1073/pnas.1604558113 27457926 PMC4995944

[37] Kim JK, Samaranayake M, and Pradhan S. Epigenetic mechanisms in mammals. Cell. Mol. Life Sci. 2009 2; 66(4): 596–612. 10.1007/s00018-008-8432-4 . 18985277 PMC2780668

[38] Guilleret I, Yan P, Grange F, Braunschweig R, Bosman FT, Benhattar J. Hypermethylation of the human telomerase catalytic subunit (hTERT) gene correlates with telomerase activity. Int J Cancer. 2002 10; 101(4): 335–41. 10.1002/ijc.10593 . 12209957

[39] Renaud S, Loukinov D, Abdullaev Z, Guilleret I, Bosman FT, Lobanenkov V, et al Dual role of DNA methylation inside and outside of CTCF-binding regions in the transcriptional regulation of the telomerase hTERT gene. Nucleic Acids Res. 2007; 35(4): 1245–56. 10.1093/nar/gkl1125 . 17267411 PMC1851636

[40] Nabilsi NH, Broaddus RR, Loose DS. DNA methylation inhibits p53-mediated survivin repression. Oncoge.2009 5; 28(19): 2046–50. 10.1038/onc.2009.62 . 19363521

[41] Wu C, Wang F, Tan L. Role and the molecular mechanism of lncRNA PTENP1 in regulating the proliferation and invasion of cervical cancer cells. Gene Ther. 2020 9 10.1038/s41434-020-00189-8 32973352

[42] Yndestad S, Austreid E, Skaftnesmo KO, Lønning PE, and Eikesdal HP. Divergent activity of the pseudogene PTENP1 in ER-positive and negative breast cancer. Divergent Activity of the Pseudogene PTENP1 in ER-Positive and Negative Breast Cancer. Mol Cancer Res. 2018 1;16(1):78–89. Pub Med 10.1158/1541-7786.MCR-17-0207 . 29021233

