# Supplementary figures and images for: Methylation of the PTENP1 pseudogene as potential epigenetic marker of age-related changes in human endometrium
Source: PLoS One. 2021 Jan 22;16(1):e0243093. doi: 10.1371/journal.pone.0243093 (PMC7822536; doi:10.1371/journal.pone.0243093)

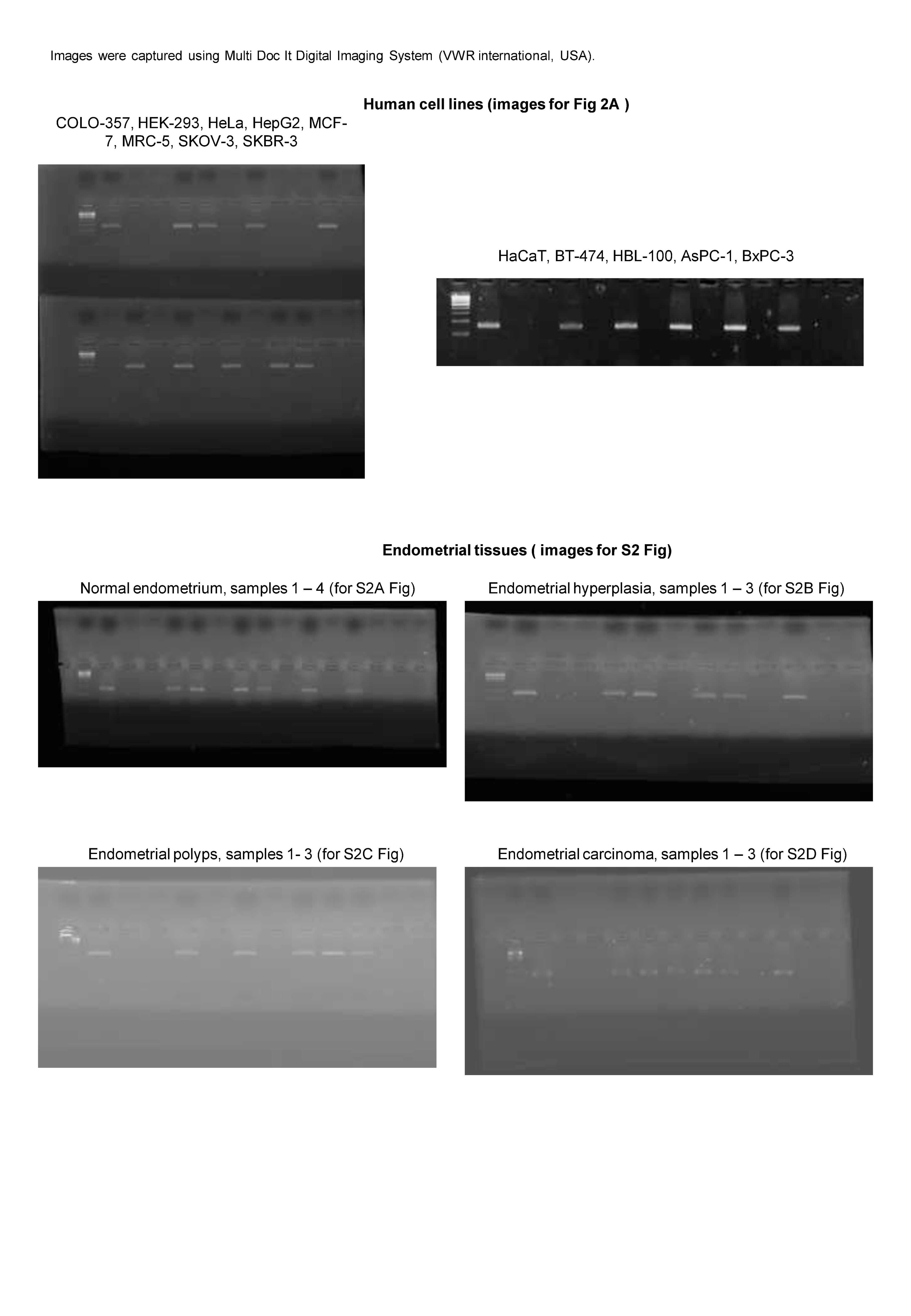

Supplement: S1 Fig — (TIF) [file pone.0243093.s001.tif]

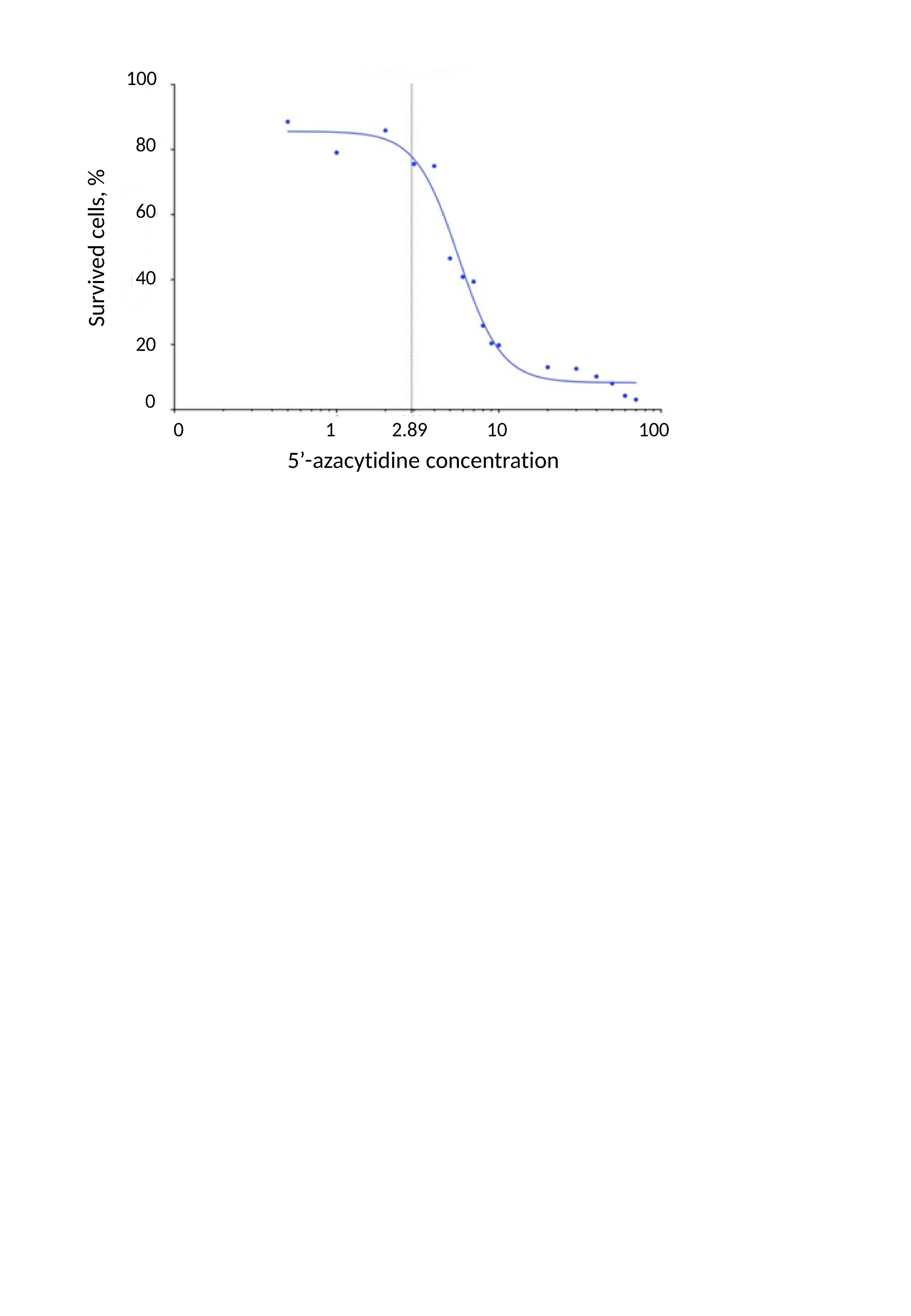

Supplement: S2 Fig — (TIF) [file pone.0243093.s002.tif]

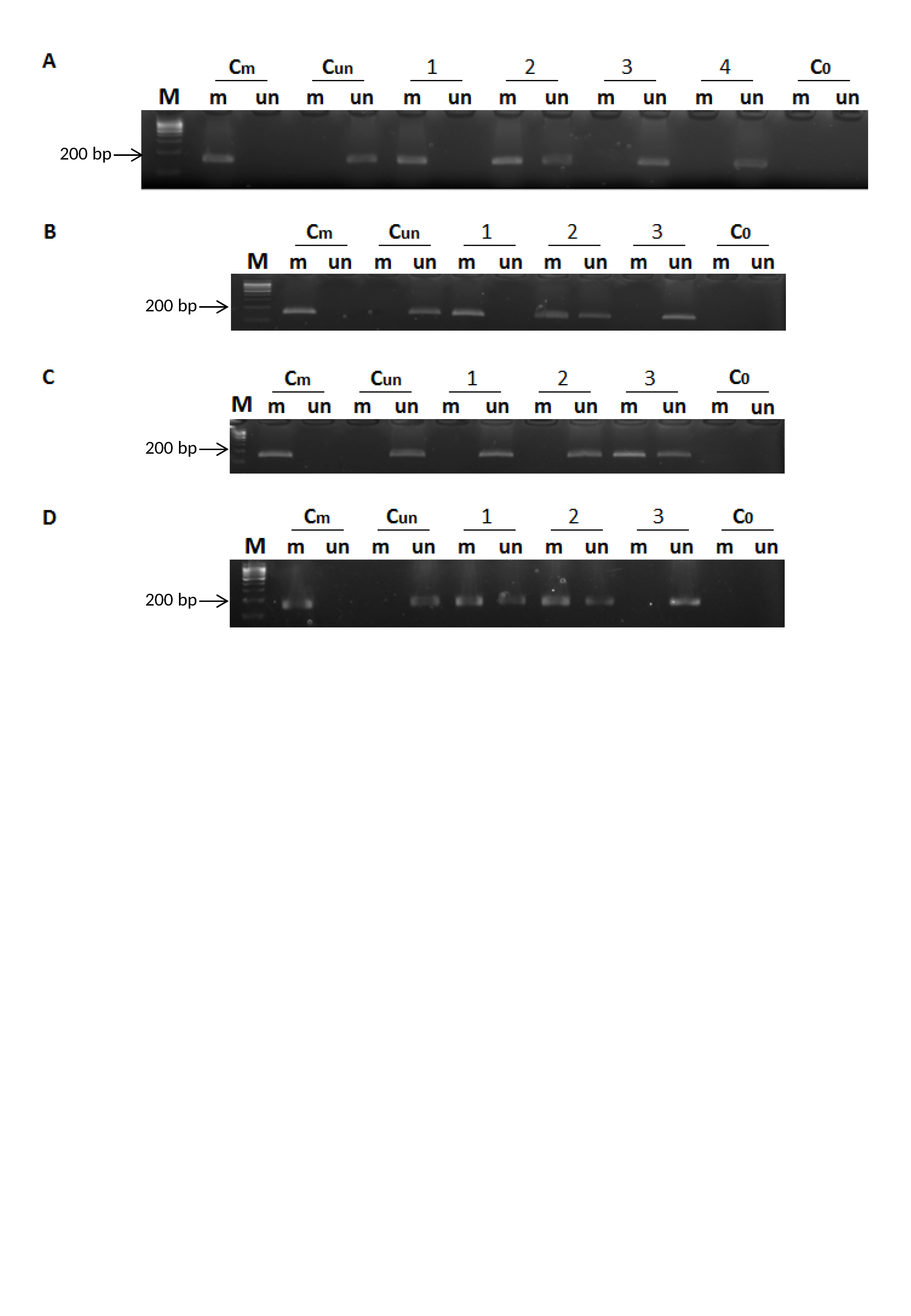

Supplement: S3 Fig — Analysis of PTENP1 methylation by MS-PCR in women with NE (A), patients with EH (B), EP (C) and EC (D). 1, 2, 3 –number of DNA samples; Cun, unmethylated control (DNA obtained from peripheral blood and treated with sodium bisulfite), Cm, methylated control (DNA obtained from peripheral blood, methylated with SssI methyltransferase, and treated with sodium bisulfite), C0, amplification without a DNA template; un and m–PCR amplification with primers for unmethylated and methylated DNA respectively; M–a molecular weight marker. (TIF) [file pone.0243093.s003.tif]

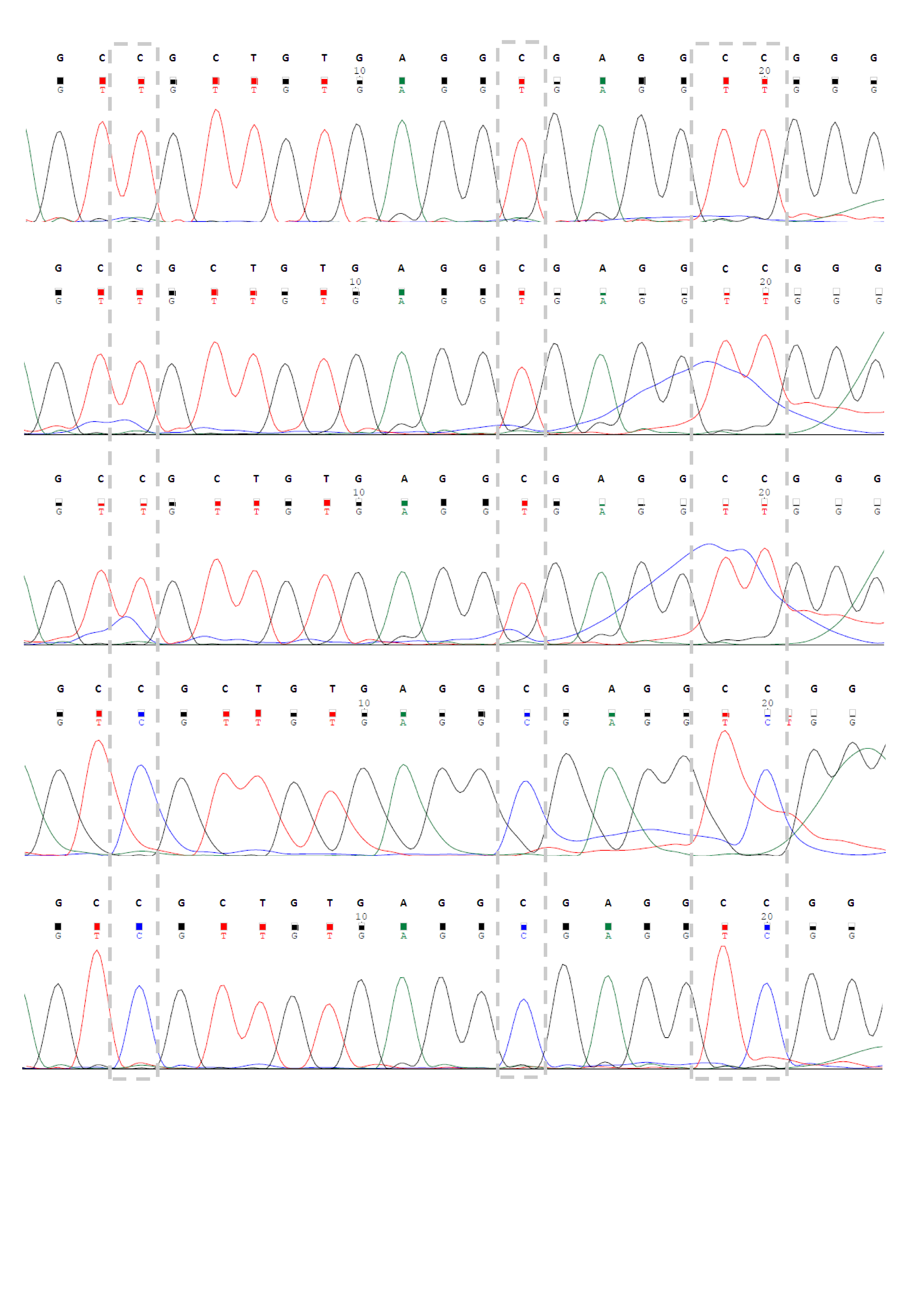

Supplement: S4 Fig — The top row of letters means the sequence of PTENP1 from the Gene Bank; the bottom row–PTENP1 sequence obtained as a result of DNA sequencing. The dotted line denotes cytosine residues that were converted in unmethylated DNA samples or remained unconverted in methylated DNA samples. (TIF) [file pone.0243093.s004.tif]

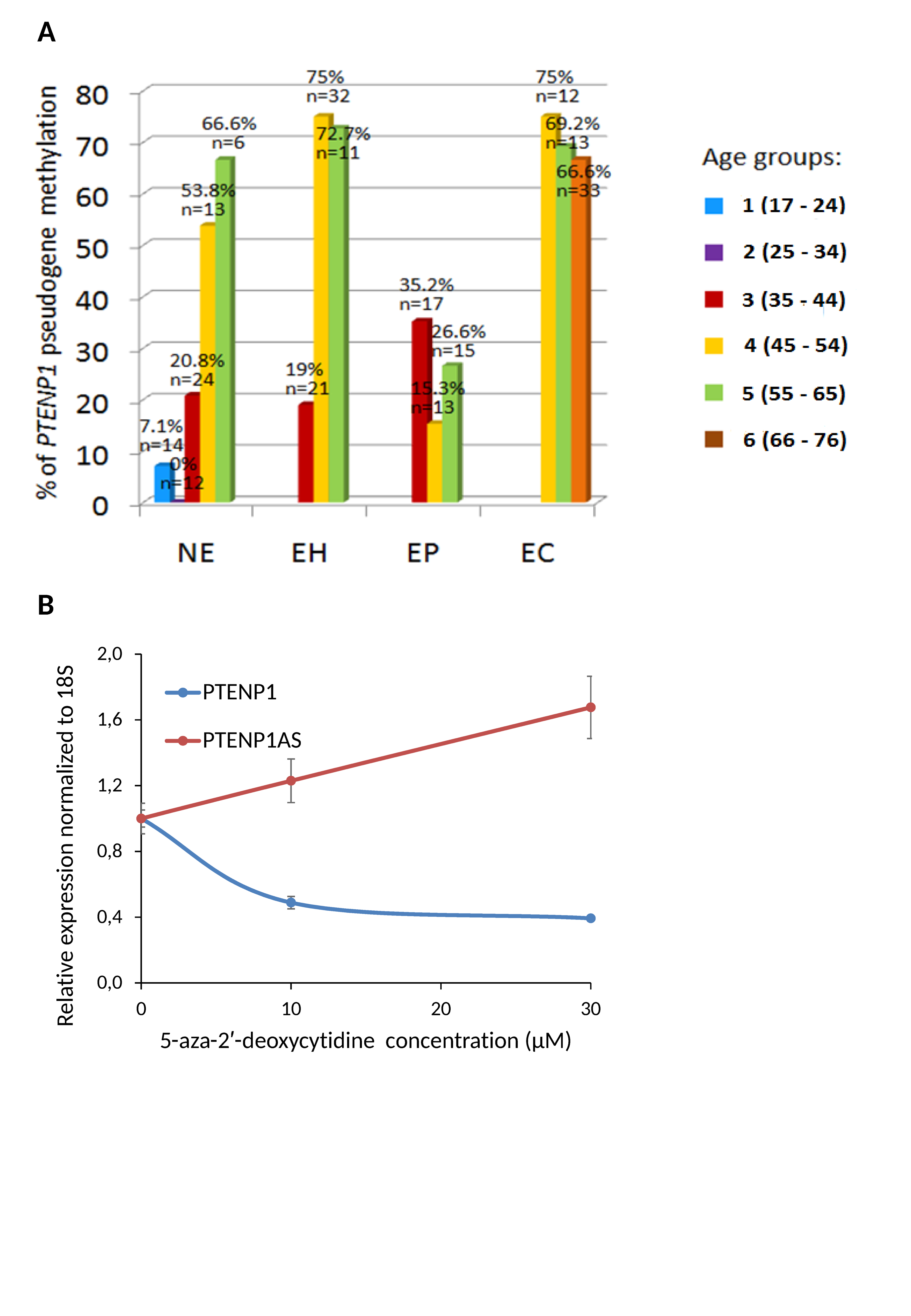

Supplement: S5 Fig — PTENP1 methylation status in different age groups of women with NE, EH, EP and EC (A); the relative levels of PTENP1 and PTENP1-AS transcripts in human glioblastoma cells, treated with 5-Aza-2′-deoxycytidine (B). DMSO treated cells were used as controls. (TIF) [file pone.0243093.s005.tif]
