# Supplementary material for: Methylation of the PTENP1 pseudogene as potential epigenetic marker of age-related changes in human endometrium
Source: PLoS One. 2021 Jan 22;16(1):e0243093. doi: 10.1371/journal.pone.0243093 (PMC7822536; doi:10.1371/journal.pone.0243093)
Supplement: S1 Table — Ta–the temperature of PCR-primer’s annealing. (DOC) [file pone.0243093.s006.doc]

| Primer | Nucleotide sequence, 5’ -> 3’ | Ta,˚С^a^ | Number of PCR-cycles | PCR-product size, bp | The source of primers |
| --- | --- | --- | --- | --- | --- |
| MS-PCR | | | | | |
| psiPN-U-F | TTGTAGTTGTGATGGAAGTTTGAAT | 64 | 33 | 156 | [25] |
| psiPN-U-R | CCACCCCCACAAATACTCACA |
| psiPN-M-F | TGTAGTCGTGATGGAAGTTTGAAT | 63 | 33 | 152 |
| psiPN-M-R | CCCCCGCGAATACTCACG |
| qRT-PCR | | | | | |
| PNP1-F | 5’- TGAAAAATCGGACGTCATCA | 62 | 45 | 124 | Developed in this study |
| PNP1-R | CTGTCCCTTATCAGATACATG |
| PNP1-AS_for | GGATGCTCACGGG | 60 | 45 | 224 |
| PNP1-AS_rev | AGGTTGGAAAGGAAAAAGTAGAACTCT |
| ACT-F | 5’-5’ CTCCTCCTGAGCGCAAGTACTC | 67 | 45 | 105 |
| ACT-R | CGGACTCGTCATACTCCTGCTT |
| PN-F | GTTTACCGGCAGCATCAAAT | 60 | 45 | 197 | [23] |
| PN-R | CCCCCACTTTAGTGCACAGT |

^a^ Primer annealing temperature
