# Supplementary material for: Methylation of the PTENP1 pseudogene as potential epigenetic marker of age-related changes in human endometrium
Source: PLoS One. 2021 Jan 22;16(1):e0243093. doi: 10.1371/journal.pone.0243093 (PMC7822536; doi:10.1371/journal.pone.0243093)
Supplement: S2 Table — (DOC) [file pone.0243093.s007.doc]

| Age groups | Number of patients; mean age ± s.d. | | | |
| --- | --- | --- | --- | --- |
|  | NE | EH | EP | EC |
| group 1  (17–24 years) | n=14;  21.5±2.2 years | - | - | - |
| group 2  (25–34 years) | n=12;  30.0±2.9 years | - | - | - |
| group 3  (35–44 years) | n=24;  40.0±2.9 years | n=21;  40.0±2.6 years | n=17;  39.0±2.9 years | - |
| group 4  (45–54 years) | n=13;  49.0±2.7 years | n=32;  49.0±2.5 years | n=13;  48.0±2.1 years | n=12;  51.5±1.8 years |
| group 5  (55–65 years) | n=6;  62.5±3.7 years | n=11;  60.0±3.2 years | n=15;  60.0±3.4 years | n=13;  59.5±2.1 years |
| group 6  (66–76) years | - | - | - | n=33;  70.0±3.2 years |
