# Supplementary material for: Methylation of the PTENP1 pseudogene as potential epigenetic marker of age-related changes in human endometrium
Source: PLoS One. 2021 Jan 22;16(1):e0243093. doi: 10.1371/journal.pone.0243093 (PMC7822536; doi:10.1371/journal.pone.0243093)
Supplement: S3 Table — (DOC) [file pone.0243093.s008.doc]

| Age groups of women with NE | **1** (17-24)  n=14  Met: 1 (7.1%) | **2** (25-34)  n=12  Met: 0 (0%) | **3** (35-44)  n=24  Met: 5 (20.8%) | **4** (45-54)  n=13  Met: 7 (53.8%) | **5** (55-65)  n=6  Met: 4 (66.6%) |
| --- | --- | --- | --- | --- | --- |
| **1** (17-24)  n=14  Met: 1 (7.1%) | - | *p*=1.000 | *p*=0.383 | *p*=0.013 | *p*=0.014 |
| **2** (25-34)  n=12  Met: 0 (0%) | *p*=1.000 | - | *p*=0.146 | *p*=0.005 | *p*=0.005 |
| **3** (35-44)  n=24  Met: 5  (20.8%) | *p*=0.383 | *p*=0.146 | - | *p*=0.067 | *p*=0.049 |
| **4** (45-54)  n=13  Met: 7  (53.8%) | *p*=0.013 | *p*=0.005 | *p*=0.067 | - | *p*=1.000 |
| **5** (55-65)  n=6  Met: 4  (66.6%) | *p*=0.014 | *p*=0.005 | *p*=0.049 | *p*=1.000 | - |
