# Supplementary material for: Methylation of the PTENP1 pseudogene as potential epigenetic marker of age-related changes in human endometrium
Source: PLoS One. 2021 Jan 22;16(1):e0243093. doi: 10.1371/journal.pone.0243093 (PMC7822536; doi:10.1371/journal.pone.0243093)
Supplement: S4 Table — (DOC) [file pone.0243093.s009.doc]

| Age groups of women with EH | **3** (35-44)  n=21  Met: 4 (19%) | **4** (45-54)  n=32  Met: 24 (75%) | **5** (55-65)  n=11  Met: 8 (72.7%) |
| --- | --- | --- | --- |
| **3** (35-44)  n=21  Met: 4 (19%) | - | *p*=0.000 | *p*=0.006 |
| **4** (45-54)  n=32  Met: 24 (75%) | *p*=0.000 | - | *p*=1.000 |
| **5** (55-65)  n=11  Met: 8 (72.7%) | *p*=0.006 | *p*=1.000 | - |
