# Supplementary material for: Methylation of the PTENP1 pseudogene as potential epigenetic marker of age-related changes in human endometrium
Source: PLoS One. 2021 Jan 22;16(1):e0243093. doi: 10.1371/journal.pone.0243093 (PMC7822536; doi:10.1371/journal.pone.0243093)
Supplement: S5 Table — (DOC) [file pone.0243093.s010.doc]

| Age groups of women with EP | **3** (35-44)  n=17  Met: 6 (35.2%) | **4** (45-54)  n=13  Met: 2 (15.3%) | **5** (55-65)  n=15  Met: 4 (26.6%) |
| --- | --- | --- | --- |
| **3** (35-44)  n=17  Met: 6 (35.2%) | - | *p*=0.407 | *p*=0.712 |
| **4** (45-54)  n=13  Met: 2 (15.3%) | *p*=0.407 | - | *p*=0.655 |
| **5** (55-65)  n=15  Met: 4 (26.6%) | *p*=0.712 | *p*=0.655 | - |
