# Supplementary material for: Methylation of the PTENP1 pseudogene as potential epigenetic marker of age-related changes in human endometrium
Source: PLoS One. 2021 Jan 22;16(1):e0243093. doi: 10.1371/journal.pone.0243093 (PMC7822536; doi:10.1371/journal.pone.0243093)
Supplement: S6 Table — (DOC) [file pone.0243093.s011.doc]

| Age groups of women with EC | **4** (45-54)  n=12  Met: 9 (75%) | **5** (55-65)  n=13  Met: 9 (69.2%) | **6** (66-76)  n=33  Met: 22 (66.6%) |
| --- | --- | --- | --- |
| **4** (45-54)  n=12  Met: 9 (75%) | - | *p*=1.000 | *p*=0.725 |
| **5** (55-65)  n=13  Met: 9 (69.2%) | *p*=1.000 | - | *p*=1.000 |
| **6** (66-76)  n=33  Met: 22 (66.6%) | *p*=0.725 | *p*=1.000 | - |
